# Supplementary material for: The Anticancer Effect of a Novel Quinoline Derivative 91b1 through Downregulation of Lumican
Source: Int J Mol Sci. 2022 Oct 29;23(21):13181. doi: 10.3390/ijms232113181 (PMC9655098; doi:10.3390/ijms232113181)
Supplement: Supplementary file 1 [file ijms-23-13181-s001.zip › ijms-1958215-supplementary.pdf]

Bio-Plex Pro Cell Signaling Assay was performed to analyse the involved signaling pathways for the treatment with compound 91b1 on KYSE150 cells. Phosphorylated analytes (AKT(Ser^{473}), ATF-2(Thr^{71}), MEK1(Ser^{217}/Ser^{221}), ErK1/2(Thr^{202}/Tyr^{204}, Thr^{185}/Tyr^{187}), p38 MAPK(Thr^{180}/Tyr^{182}), HSP27(Ser^{78}), p53(Ser^{15}), JNK(Thr^{183}/Tyr^{185}), p90 RSK(Ser^{380}), and Stat 3(Ser^{727}) from cell lysates treated with gradually increased concentrations of compound 91b1 (5, 9.5, and 20μg/mL) or vehicle control were detected by Bio-Rad Bio-Plex 200 Suspension Array System. Figure S1. shows the summary of phosphorylated analytes from compound 91b1 treated KYSE150 cells.

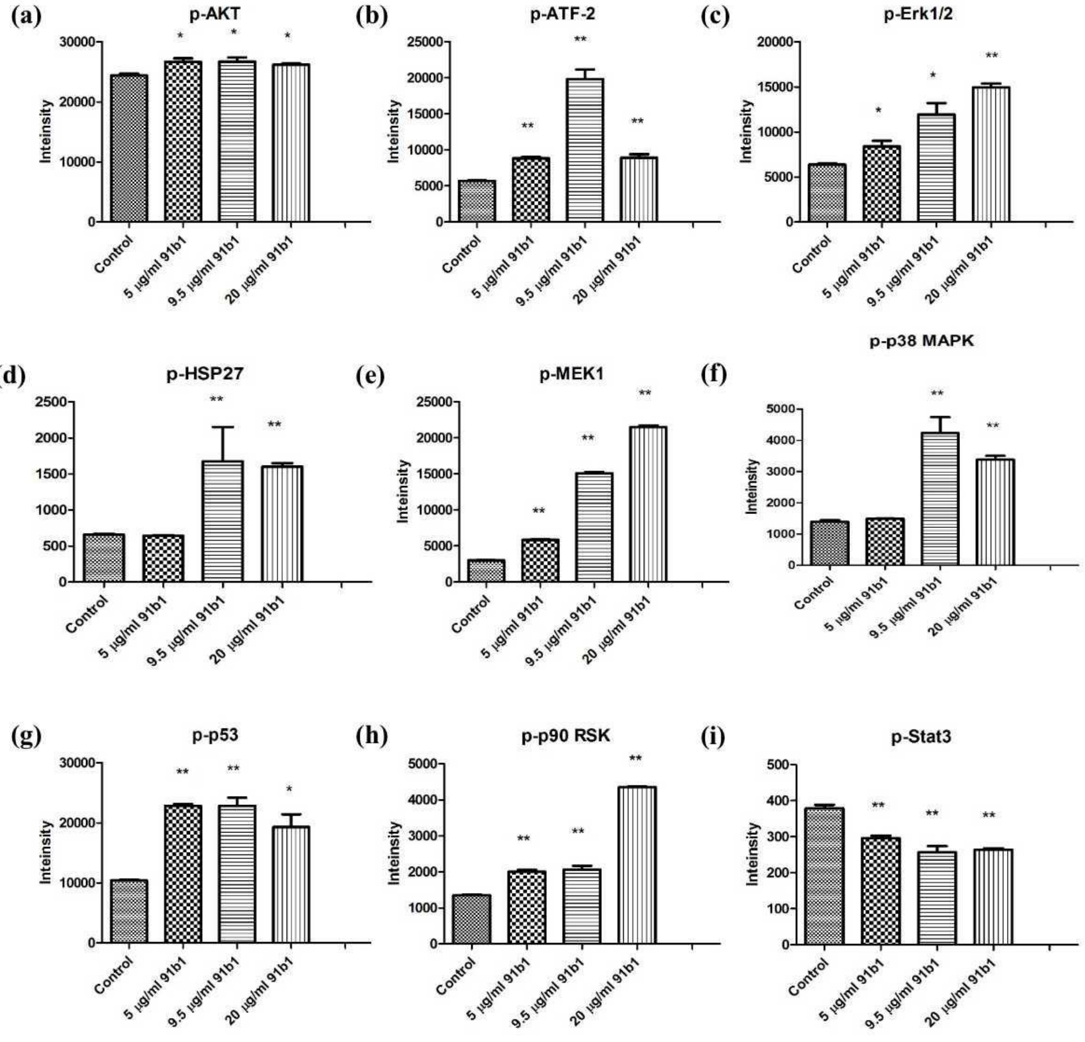

Figure S1. Phosphorylated analytes from KYSE150 cells treated with different concentrations of compound 91b1 or vehicle control analyzed by Bio-plex 200. (A): Phosphorylated AKT(Ser^{473}); (B): Phosphorylated ATF-2 (Thr71); (C): Phosphorylated ErK1/2 (Thr202/Tyr204, Thr185/Tyr187); (D): Phosphorylated HSP27 (Ser78); (E): Phosphorylated MEK1 (Ser217/Ser221); (F): Phosphorylated p38 MAPK (Thr180/Tyr182); (G): Phosphorylated p53 (Ser15); (H): Phosphorylated p90 RSK (Ser380) ; (I):Phosphorylated Stat 3 (Ser727). Compound 91b1 concentrations ranged from5, 9.5, and 20μg/mL. 0.1% DMSO was applied as the vehicle control. N=3. * $p<0.05$; ** $p<0.01$.

According to the results, the levels of phosphorylated AKT, ATF-2, ErK1/2, HSP27, MEK1, p38 MAPK, p53, and p90 RSK were increased significantly after treated with compound 91b1 and showed a dose-depend manner except for 20μg/mL in some groups (p-ATF-2, p-p38 MAPK, and p-p53 level after treated with 20μg/mL compound 91b1 were lower than those treated with 10μg/mL compound 91b1, but were still increased significantly than control). Phosphorylated Stat3 of cell lysates was decreased significantly as the concentrations of compound 91b1 were gradually increased.

LC/MS Report

File D:\DATA\A02\MONITOR\NS-03-07931-LCMSA034.D
Injection Date : 16 Jun 14 3:57 pm +0800 Tgt Mass(EZX) :
Sample Name : NS-03 Location : P1-B-07
Acq. Operator : A02-Monitor Inj : 1
Spec. Reported : MS Integration Inj Volume : 0.1 ul
Acq. Method : D:\METHODS\1-POS-MON.M
Analysis Method : D:\METHODS\1-POS-MON.M
Sample Info : Easy-Access Method: '1-POS-MON'
Method Info : Mobile Phase: A: water(0.01%TFA) B: ACN(0.01%TFA)
Gradient: 5%-95% B in 1.3min
Flow Rate: 2ml/min
Column: Sunfire C18, 4.6*50mm,3.5um
Oven Temperature: 50 C
MS" ESI-POS Mass 103-1000

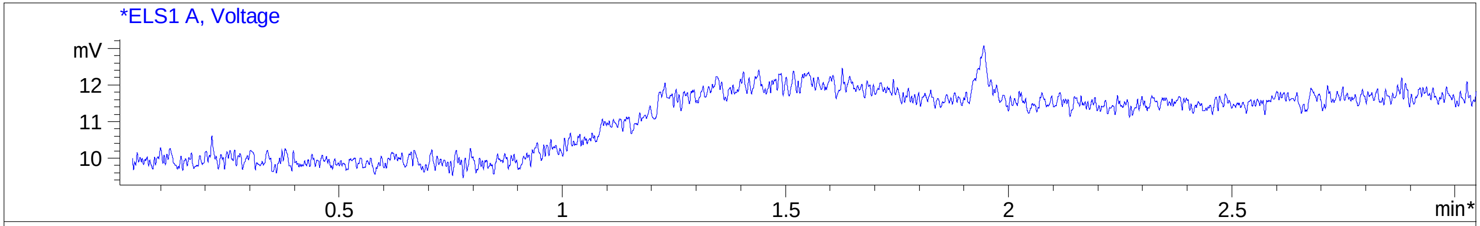

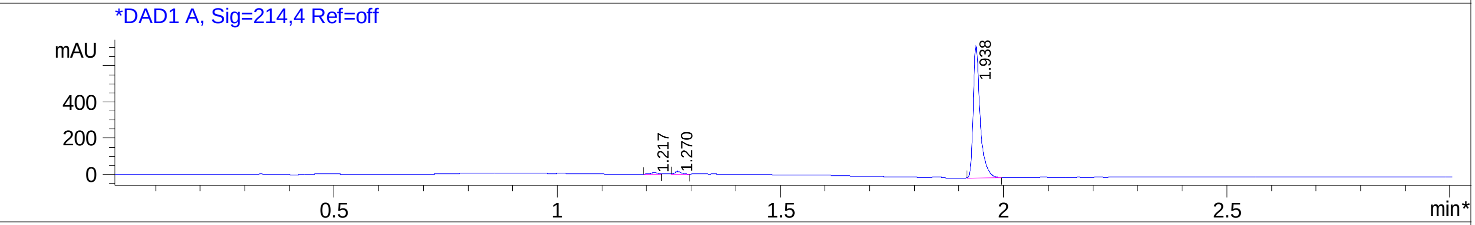

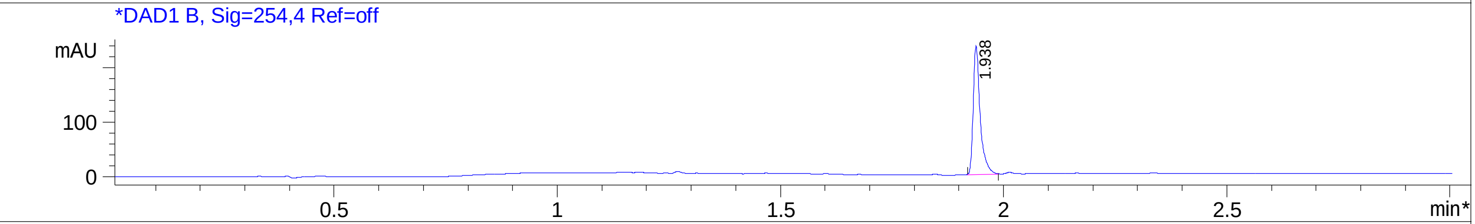

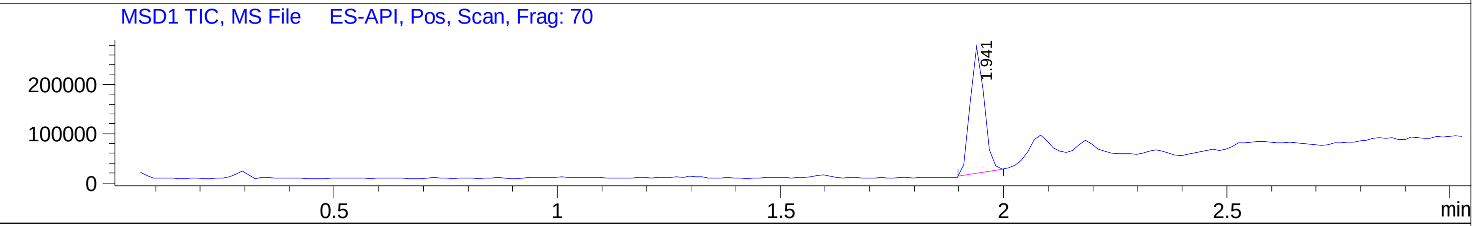

Integration Results for ELS1 A, Voltage

RetTim	Width	Area	Height	Area%

Integration Results for DAD1 A, Sig=214,4 Ref=off

RetTim	Width	Area	Height	Area%
1.22	0.02	10.90	9.69	1.34
1.27	0.01	14.35	14.89	1.77
1.94	0.02	787.02	728.39	96.89

Integration Results for DAD1 B, Sig=254,4 Ref=off

RetTim	Width	Area	Height	Area%
1.94	0.02	257.80	236.69	100.00

Ret. Time: 1.94 <<<< POSITIVE SPECTRA >>>>

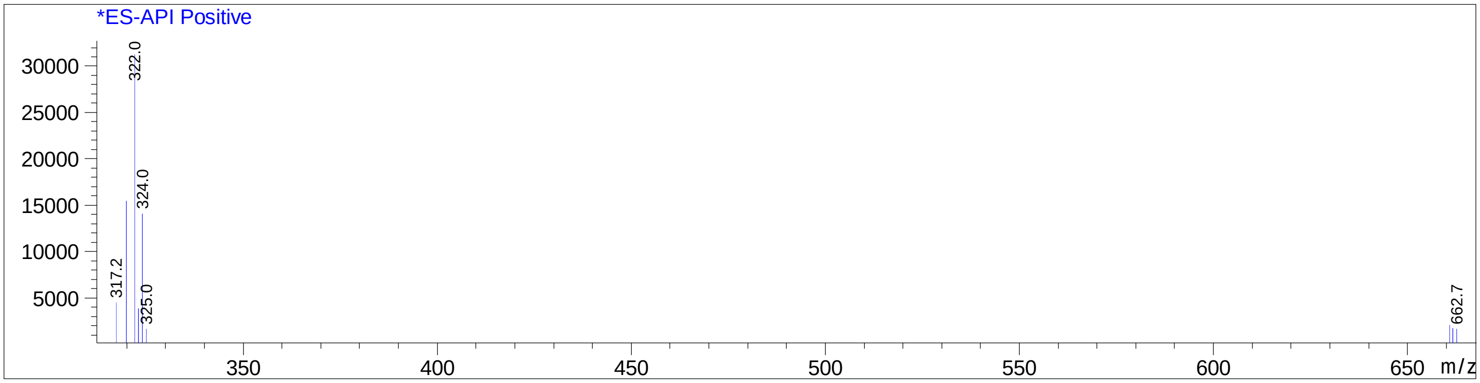

Figure S2. LC/MS report of compound 91b1.
